# How does indirect air-cooling influence pulp chamber temperature in different volume teeth and absence/presence of resin-based composite during light curing?

**DOI:** 10.1186/s12903-022-02545-z

**Published:** 2022-11-24

**Authors:** Mathieu Mouhat, Lina Stangvaltaite-Mouhat, Emil Finnäs, Amani Andersen, Anneli Lirhus Evertsen, Bo W. Nilsen

**Affiliations:** 1 Department of Clinical Dentistry, Faculty of Health Sciences, UiT The Arctic University of Norway, Tromsø, Norway; 2 Oral Health Centre of Expertise in Eastern Norway, Sørkedalsveien 10A, 0369 Oslo, Norway

**Keywords:** LED dental curing light, Pulp chamber, Temperature, Composite resins, Dentistry

## Abstract

**Background:**

Light-curing of materials during restorative dental procedures poses a risk for pulp tissue overheating. Therefore, the aim of this study was to investigate the effect of indirect air-cooling on pulp chamber temperatures during light-curing of varying volume teeth and absence/presence of resin-based composite (RBC) at different exposure time.

**Methods:**

The volume of 11 human teeth was measured by micro computed tomograph. An experimental rig controlled the thermal environment of the teeth and a thermocouple inserted retrograde into the root canal measured temperature changes. Pulp chamber temperature was measured with and without air-cooling on teeth without and with RBC at 15 s, 30 s and 60 s intervals. Generalized estimating equations were used for statistical analysis.

**Results:**

The temperature increase with air-cooling (versus no air-cooling) was lower in teeth despite absence/presence of RBC (β = − 4.26, 95%CI − 5.33 and β = − 4.47, 95%CI − 5.60, respectively). With air-cooling, the temperature increase in teeth with RBC was lower compared to teeth without RBC (β = − 0.42, 95%CI -0.79; − 0.05). Higher teeth volume resulted in lower temperature increase with air-cooling than without air-cooling (β = − 0.04, 95%CI -0.07; − 0.01 and β = − 0.17, 95%CI -0.30; − 0.05, respectively).

**Conclusions:**

Air-cooling resulted in lower pulp chamber temperature increase. Using air-cooling, the temperature increase was lower in teeth with RBC compared to teeth without RBC. Lower volume teeth resulted in higher temperature increase, thus they seemed to benefit more from air-cooling compared to higher volume teeth. Air-cooling could be an effective tool in controlling pulp temperature increase during light-curing, especially when the tooth volume is small.

## Background

In restorative dentistry, light curing of adhesives and resin-based composites (RBCs) with light-emitting diode (LED) light-curing units (LCUs) generates heat, which can be potentially harmful for the pulp and adjacent soft tissues [1, 2]. More recently, LED-LCUs with higher radiant emittance, up to 3000 mW/cm^2^, have been commercialized [3, 4]. Higher radiant emittance of the LED-LCU has been shown to result in the pulp chamber temperature increase [5, 6]. The pulp tissues have previously been reported to tolerate a temperature increase of between 5.5 °C and 11 °C [7, 8], while in vitro studies have reported a pulp chamber temperature increase ranging from 1.5 °C to 23.2 °C when using LED-LCUs [6, 9–15]. A recent in vivo study confirmed the previous in vitro findings that higher radiant emittance is responsible for higher pulp temperature rise [16]. Since pulp temperature increase during light curing may be a cause of pulp injury it might be necessary to consider measures that help control heat generation to prevent iatrogenic pulp damage during restorative procedures [17, 18].

Different strategies have been suggested to reduce heat development during the polymerization of RBC such as LED-LCU featuring discontinuous output mode [19, 20] or decreased irradiance [21]. But these two approaches may lead to sub-optimal monomer conversion and decrease the mechanical properties of RBC [22]. Air-cooling by the three-way syringe is another method that can be used to reduce the thermal strain on the pulp during light-curing with LED-LCUs; it has been suggested that air-cooling is beneficial, especially during long exposure times and when using high radiant emittance LED-LCUs [23], although the scientific data is limited. A previous work investigated air-cooling, water and water spray during luting of onlays concluded that the application of air-cooling during the LCU irradiation is the most effective method to reduce temperature rise; the pulp temperature was decreased by 4 °C with air-cooling procedures compared to no cooling procedure when using a LED-LCU on a mandibular extracted human molar [24]. Another study showed that a direct air-cooling system embedded in an experimental LED-LCU, which blew air constantly in the direction of the LED-LCU tip was effective in decreasing the maximum pulp temperature increase in one premolar [13]. Zarpellon and co-workers investigated the in vivo pulp temperature rise in premolars (*n* = 9) when using a wide-spectrum LED-LCU and showed that air-cooling application prevented pulp temperature increase in premolars during LED-LCU exposure. Interestingly, when air-cooling was applied for 30 s simultaneously with LED-LCU exposure, the peak temperature was reported to be 34 °C while the baseline temperature was 35.4 °C [25]. Clearly, air-cooling has a potential to be an effective tool to manage pulp temperature increase. Nonetheless, this study was performed only on premolars. The volume of coronal hard tissues varies between teeth, i.e.*,* between a molar, a premolar and an incisor, and it is unclear if similar results could be shown in teeth with different volume. The amount of tooth substance might affect the tooth’s ability to dissipate heat generated during light curing of restorative materials [26]. It has recently been shown that the presence of RBC might to some extent promote thermal insulation of the pulp chamber during LED-LCU exposure [15, 27, 28]. To the best of the authors’ knowledge, there is no previously published work investigating pulp chamber temperature development in teeth with different volume, without and with RBC, during LED-LCU exposure along with application of an air-cooling.

Therefore, the aim of this study was to evaluate the effectiveness of indirect air-cooling on pulp chamber temperature changes during light-curing of teeth with and without RBC and varying volume at different exposure time.

In line with the aim, the following working hypotheses were established:

(i) Temperature increase in pulp chamber is higher without air-cooling compared to air-cooling during LED-LCU exposure.
(ii) There is a difference in pulp chamber temperature increase in teeth with RBC and without RBC with air-cooling during LED-LCU exposure.
(iii) There is an association between coronal tooth hard tissue volume and pulp chamber temperature increase during LED-LCU exposure.

## Methods

### Preparation of teeth

Eleven caries-free, extracted human teeth supplied by the surgical department at the Oral Health Centre of Expertise in Northern Norway were used. There were six upper 3rd molars, three lower 3rd molars and two lower premolars. The teeth were stored in 0.5% Chloramine-T solution according to ISO/TS 11405–2015 in a refrigerator (4 ± 1 °C) prior to use and in-between the experiments. The root canals were prepared retrograde with endodontic files (K-file Nitiflex, Dentsply Sirona, Charlotte, NC, USA) up to size ISO # 70 leaving a layer of dentine between the pulp chamber and a class 1 cavity. The calibrated thermocouple (Type T copper constantan, Omega Engineering, Manchester, UK) was inserted into the pulp chamber as close to the buccal pulp horn as possible under the radiographic control. The thermocouple was fixed using GC Fuji I Glass Ionomer luting cement (GC Corporation, Tokyo, Japan).

### LED-LCU and three-way syringe

In this study we used a wide-spectrum LED-LCU (Bluephase G2® Ivoclar Vivadent, Schaan, Liechtenstein) in high mode (≈ 1400 mW/cm^2^). The irradiance was controlled with a calibrated laboratory-grade NIST-references USB4000 spectrometer (Managing Accurate Resin Curing (MARC) System; Bluelight Analytics Inc., Halifax, Canada) prior to- and after each testing session.

The three-way syringe was placed 2 cm from-, and perpendicular to the lingual surface of the tooth. The average airflow provided by the three-way syringe was assessed with a TA5 Anemometer (Airflow, Rheinbach, Germany). Prior to the experiments, the airflow of 21 three-way syringes at the University Dental Clinic was measured to establish a reference for the experiments. The average airflow was determined to be 31.4 m/s. For the study, the airflow of the three-way syringe was measured before and after the experiments. On average the airflow was 31.6 m/s.

### Setup of the experiments

To simulate the environmental conditions within the oral cavity, the teeth were inserted in a suitably sized hole cut in a thin plastic sheet, with the root protruding out on one side of the hole and the coronal part on the opposite side. The plastic sheet with the teeth was placed in a thermostatically controlled and circulated water bath (AH15L HT, Avantor, Radnor, PA, USA) maintained at 37 ± 1 °C with the root being immersed in the water up to the level of the cemento-enamel junction and the coronal part in the air. The baseline temperature in the pulp chamber was established by keeping the tooth in the water bath for 10 min prior to testing, allowing the pulp temperature to stabilize. Pulp chamber temperature changes were continuously recorded with a data logger (OQ610 temperature logger, Grant instruments, Cambridge, UK) using the software SquirrelView (version 3.9, Grant instruments, Cambridge, UK) connected to a standard desk-top computer.

A sensor (Fisherbrand™ Traceable™ Humidity Meter, Thermo Fischer Scientific, Waltham, MA, USA) was used to verify the room temperature and relative humidity between test cycles.

The total dentine and enamel volume of the coronal parts of the teeth used in the experiment were measured with a micro computed tomograph (Micro-CT) (Bruker Skyscan 1272, Bruker, Kontich, Belgium). The Micro-CT scanning was performed at an iso-tropic resolution of ~ 12 mm/voxel and the projections were reconstructed with filtered back-projection, using the software NRecon (Bruker, Kontich, Belgium). Volume calculations were carried out with the analyse software CTAn (Bruker, Kontich, Belgium). The position of the thermocouple in relation to the LCU, and the thickness of the dentin and enamel, was measured perpendicular to the long-axis of the teeth in Dataviewer software (Bruker, Kontich, Belgium).

### Protocol of the experiments

Two protocols were run, one for teeth having class 1 cavity without RBC (without RBC) and one for teeth having class 1 cavity with RBC (with RBC) (Tetric EvoFlow® A3, Ivoclar Vivadent, Schaan, Liechtenstein). Based on pre-study pilots (involving four repeated measurements in the same tooth), it was determined that one measurement per tooth were appropriate due to the large mean differences observed between the groups with and without air-blowing, and because a relative small coefficient of variation between the experimental groups (ranging from 0.5 - 3.6% in groups without air-cooling, to 0.4–3.8% in groups with air-cooling).

The cavities were prepared in a standardized way. The protocol was designed to simulate the clinical procedure of air drying, dentine priming and bonding, light curing of the bonding agent and RBC placement prior to the final light curing process, in accordance with the dry-bonding technique. The test cycles were run with and without indirect air-cooling for each sample, resulting in four data sets per sample.

For the teeth without RBC the protocol was as follows: 10 s of air-drying for removal of excess moisture, at a 45 ° downward angle on the lingual surface; the cavity was confirmed to be dry; 60 s waiting time to simulate the application of the primer- and bonding agent; LED-LCU exposure, separate measurements of pulp chamber temperature at 0 s, 15 s, 30 s and 60 s [29]. The tip of LED-LCU was placed at 1 mm from the surface of the tooth.

For the teeth with RBC the protocol was as follows: 10 s of air-drying for removal of excess moisture, at a 45 ° downward angle at the lingual surface; 30 s of waiting to simulate the application of the primer- and bonding agent; 10 s of LED-LCU exposure (to simulate curing of the bonding agent), a thin layer of glycerol was applied to the cavity (for the cavity to be re-used), an application of RBC directly into the cavity (a new portion of RBC was applied before 15 s, 30 s and 60 s measurements); LED-LCU exposure, separate measurements of pulp chamber temperature at 0 s, 15 s, 30 s and 60 s [29]. The cured RBCs were removed between the measurements and a new portion of uncured RBC was placed. The tip of LED-LCU was placed at 1 mm from the surface of the tooth.

### Ethical permission

Since the study involved the use of extracted human teeth, ethical permission was asked for from the Norwegian Regional committee for Medical and Health Research Ethics (REC). They concluded that such permission was not necessary (2015/234/REK Nord).

### Statistics

Graphs and statistical analyses were performed using Sigmaplot 14 (Systat. Software, San Jose, CA, USA) and Statistical Package for the Social Sciences (SPSS, Version 28.0, IBM, Armonk, NY, USA).

Univariable generalized estimating equations were used to investigate the association between presence/absence of air-cooling, presence/absence of RBC, volume of the teeth, distance between LED-LCU tip and thermocouple tip, and the temperature change in pulp chamber. The analyses included LED-LCU exposure times of 15 s, 30 s, 60 s. Multivariable analysis could not be performed due to small sample size. The significance was set at *p* < 0.050. β values are presented with robust 95% confidence intervals (CI).

## Results

### Temperature increase in pulp chamber with and without air-cooling during LED-LCU exposure

**Fig. 1:**
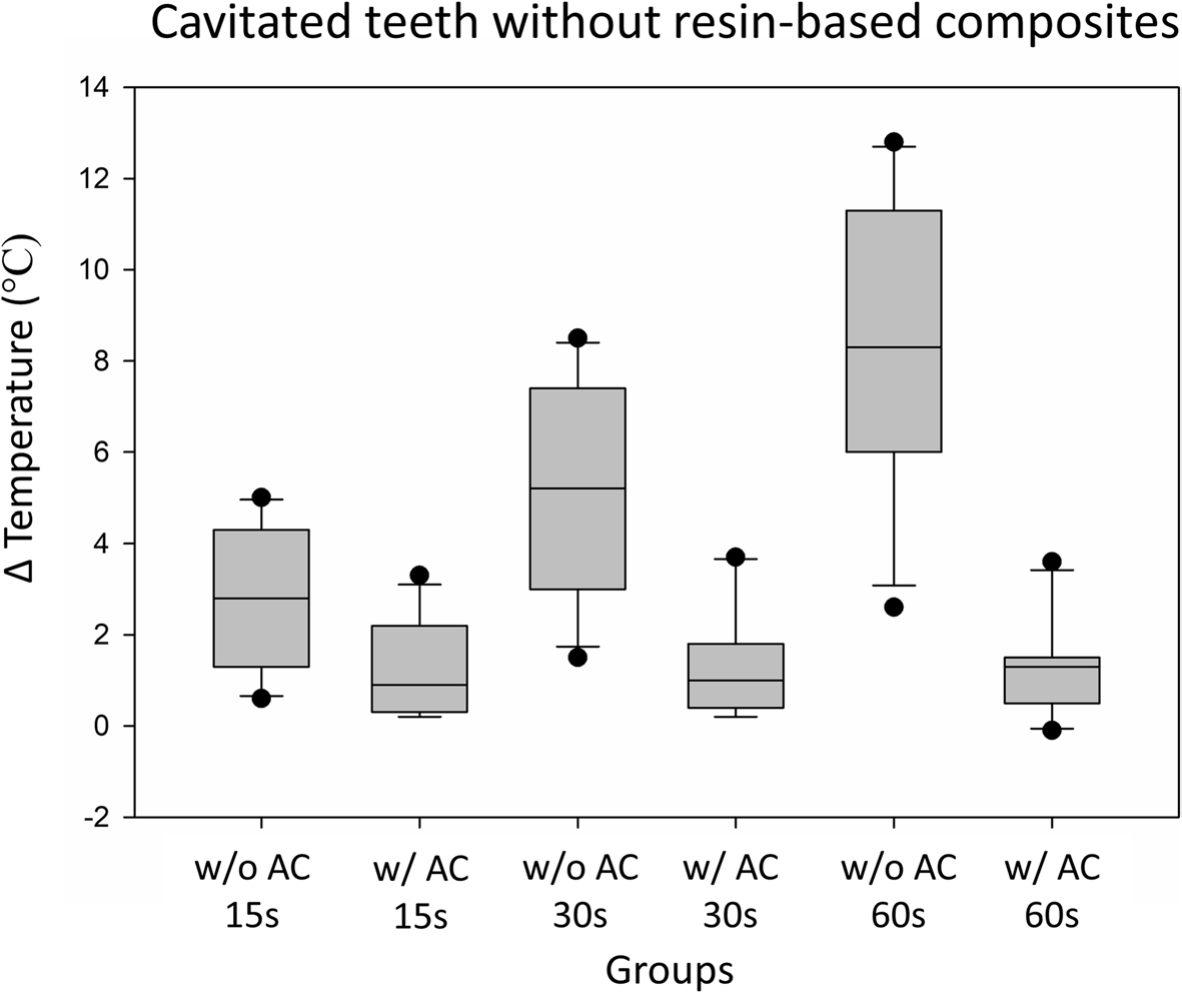
Median increase in pulp chamber temperature during LED-LCU exposure for the cavitated teeth without resin-based composite (RBC) (*n* = 11). w/o AC = without air-cooling and w/ AC = with air-cooling at 15, 30 and 60 s. Grey box represent the Q1-Q3. Black line within box represent median. Whiskers represent the minimum and maximum value (calculated as Q1–1.5*IQR or Q3 + 1.5*IQR). Black circles represent outliers (Values that fall above or below the IQR, > Q3 + 1.5*IQR and is < Q1–1.5*IQR)

The median temperature increase in the pulp chamber after 30 s of LED-LCU exposure in teeth without RBC and without air-cooling was 5.2 °C (interquartile range (IQR) 3.0–7.4), while with air-cooling it was 1 °C (IQR 0.4–1.8) (Table 1 and Fig.1).

**Table 1:** Description of the teeth investigated. Temperature changes within the pulp chamber at 15-, 30- and 60-seconds exposure time for teeth with/without resin-based composites (RBC) and with/without indirect air-cooling (AC) applied

Teeth #	Total volume (mm^**3**^)	Volume enamel (mm^**3**^)	Volume dentine (mm^**3**^)	VolumePulp (mm^**3**^)	Distance between the LCU and thermocouple (mm)	Dentin thickness (mm)	Enamel thickness (mm)	Total thickness (mm)	Δ Temperature teeth without RBC without air cooling (°C)			Δ Temperature teeth without RBC with air cooling (°C)			Δ Temperature teeth with RBC without air cooling (°C)			Δ Temperature teeth with RBC with air cooling (°C)		
									15 s	30s	60s	15 s	30s	60s	15 s	30s	60s	15 s	30s	60s
1	451.95	196.21	247.88	10.81	3.56	2,03	0	2,03	0.6	1.5	2.6	0.2	0.2	0.1	0.4	1.3	2.6	0.1	−0.1	−0.5
2	345.94	181.77	158.19	10.42	3.59	2,33	0,26	2,59	5.0	8.5	12.8	2.3	3.5	1.5	5.9	9.1	12.5	1.5	0.0	0.2
3	407.69	212.24	188.27	12.61	4.14	2,82	0,32	3,14	4.3	7.3	10.5	1.6	1.8	2.7	3.7	6.1	8.4	2.5	2.4	2.1
4	488.32	171.96	307.64	7.34	5.19	3,17	0	3,17	0.9	3.0	6.3	0.9	1.1	1.2	1.3	3.0	5.2	0.5	0.4	−0.2
5	485.47	213.64	263.94	10.06	4.52	2,7	0,82	3,52	1.3	2.7	5.0	0.8	1.0	1.3	1.0	2.2	4.0	0.5	0.0	−0.2
6	663.08	266.93	384.88	12.56	4.65	3,01	0,64	3,65	1.3	3.2	6.0	0.3	0.4	1.3	1.4	3.3	6.2	0.0	0.4	1.1
7	406.62	194.30	205.47	8.86	4.0	2,62	0,38	3	2.8	5.0	7.4	0.8	0.8	1.3	3.0	5.3	7.9	1.2	0.2	0.0
8	386.61	198.45	182.06	7.86	4.17	2,72	0,45	3,17	4.3	7.4	11.3	3.3	3.7	3.6	4.8	7.5	10.6	2.8	2.6	3.4
9	380.16	188.89	184.27	4.81	3.75	2,05	0	2,05	2.8	5.2	8.3	0.2	0.2	−0.1	3.2	5.4	8.3	0.7	−0.3	−0.8
10	322.33	154.51	162.09	7.96	4.49	2,43	1,06	3,49	4.8	8.0	12.3	2.2	1.7	0.5	4.8	7.5	11.2	2.2	1.6	2.3
11	332.30	167.05	158.91	6.69	2.89	1,79	0	1,79	4.0	6.7	10.2	1.2	0.4	0.9	4.0	6.4	9.4	1.4	0.6	0.4
**Median (inter quartile range (IQR))**									**2.8 (1.3–4.3)**	**5.2 (3.0–7.4)**	**8.3 (6.0–11.3)**	**0.9 (0.3–2.2)**	**1.0 (0.4–1.8)**	**1.3 (0.5–1.5)**	**3.2 (1.3–4.5)**	**5.4 (1.7–2.7)**	**8.3 (5.2–10.6)**	**1.2 (0.5–2.2)**	**0.4 (0.0–1.6)**	**0.2 (0.2–2.1)**

In teeth without RBC, the temperature increase in pulp chamber with air-cooling was 4.26 °C lower compared to no air-cooling (β = − 4.26, 95%CI -5.33; − 3.18) (Table 2).

**Table 2:** Influence of air-cooling (AC), resin-based composite (RBC), volume of teeth (volume) and distance between the pulpal wall and the tip of the thermocouple (distance) on pulp chamber temperature increase according to generalized estimating equations. β coefficients, robust 95% confidence intervals and*p* values for AC effect are presented stratified by RBC, for RBC effect stratified by AC and pooled for volume and distance effect

Factors	Resin-based composite		Air-cooling		Pooled
	No	Yes	No	Yes	
	β (95% CI) *p* value				
AC					
*No*	Ref.	Ref.	–	–	–
*Yes*	−4.26 (−5.38; −3.18) < 0.001	−4.47 (−5.60; −3.34) < 0.001	–	–	–
RBC					
*No*	–	–	Ref.	Ref.	–
*Yes*	–	–	−0.20 (−0.48; −0.08) 0.152	−0.42 (−0.79; −0.05) 0.026	–
Volume * Cont.*	−0.10 (− 0.17; − 0.04) 0.002	−0.10 (− 0.18; − 0.03) 0.010	−0.17 (− 0.30; − 0.05) 0.006	−0.04 (− 0.07; − 0.01) 0.022	−0.10 (− 0.18; − 0.03) 0.005
Distance * Cont.*	–	–	–	–	− 0.50 (−1.59; 0.59) 0.366

*Ref.* reference category, *Cont.* continuous

**Fig. 2:**
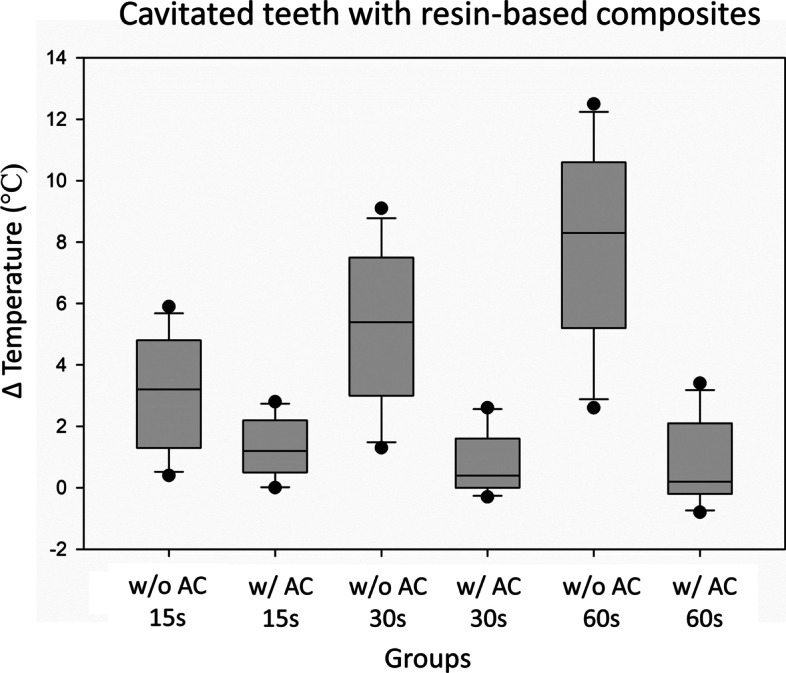
Median increase in pulp chamber temperature during LED-LCU exposure for the cavitated teeth with resin-based composite (RBC) within the cavity (*n* = 11). w/o AC = without air-cooling and w/ AC = with air-cooling at 15, 30 and 60 s. Grey box represent the Q1-Q3. Black line within box represent median. Whiskers represent the minimum and maximum value (calculated as Q1–1.5*IQR or Q3 + 1.5*IQR). Black circles represent outliers (Values that fall above or below the IQR, > Q3 + 1.5*IQR and is < Q1–1.5*IQR)

The median temperature in the pulp chamber after 30 s of LED-LCU exposure in teeth with RBC and without air-cooling was 5.4 °C (IQR 1.7–2.7), while with air-cooling 0.4 °C (IQR -0.0-1.6) (Table 1 and Fig.2).

In teeth with RBC, the temperature increase in pulp chamber with air-cooling was 4.47 °C lower than in teeth without air-cooling (β = − 4.47, 95%CI -5.60; − 3.34) (Table 2).

### Pulp chamber temperature increase in teeth with RBC and without RBC with air-cooling during LED-LCU exposure

With air-cooling, the temperature increase in pulp chamber in teeth with RBC was 0.42 °C lower compared to teeth without RBC (β = − 0.42, 95%CI -0.79; − 0.05) (Table 2).

### Volume of the teeth and pulp chamber temperature increase during LED-LCU exposure

**Fig. 3:**
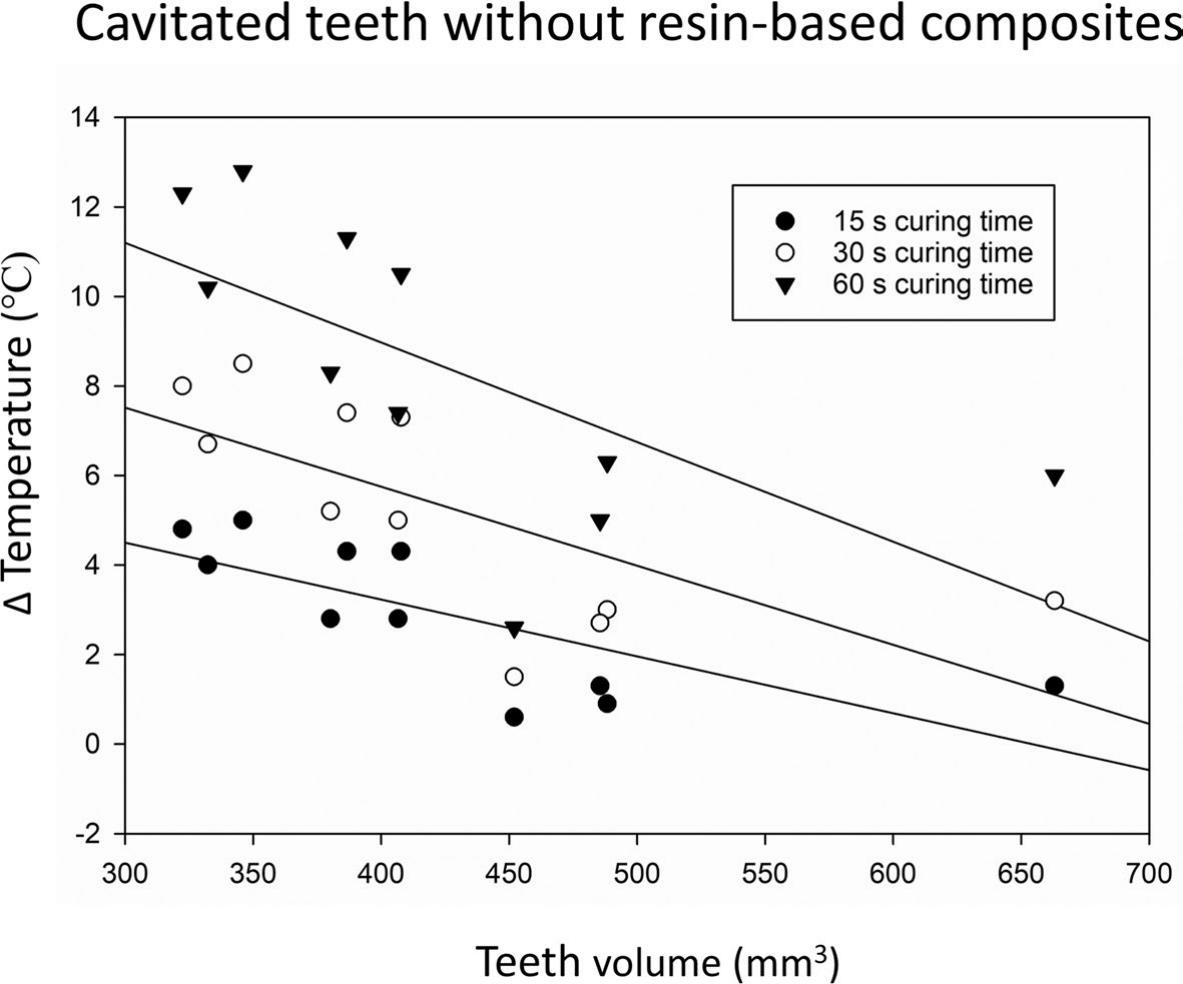
Relationship between pulp chamber temperature increase and the tooth volume for the teeth without resin-based composite (RBC)

**Fig. 4:**
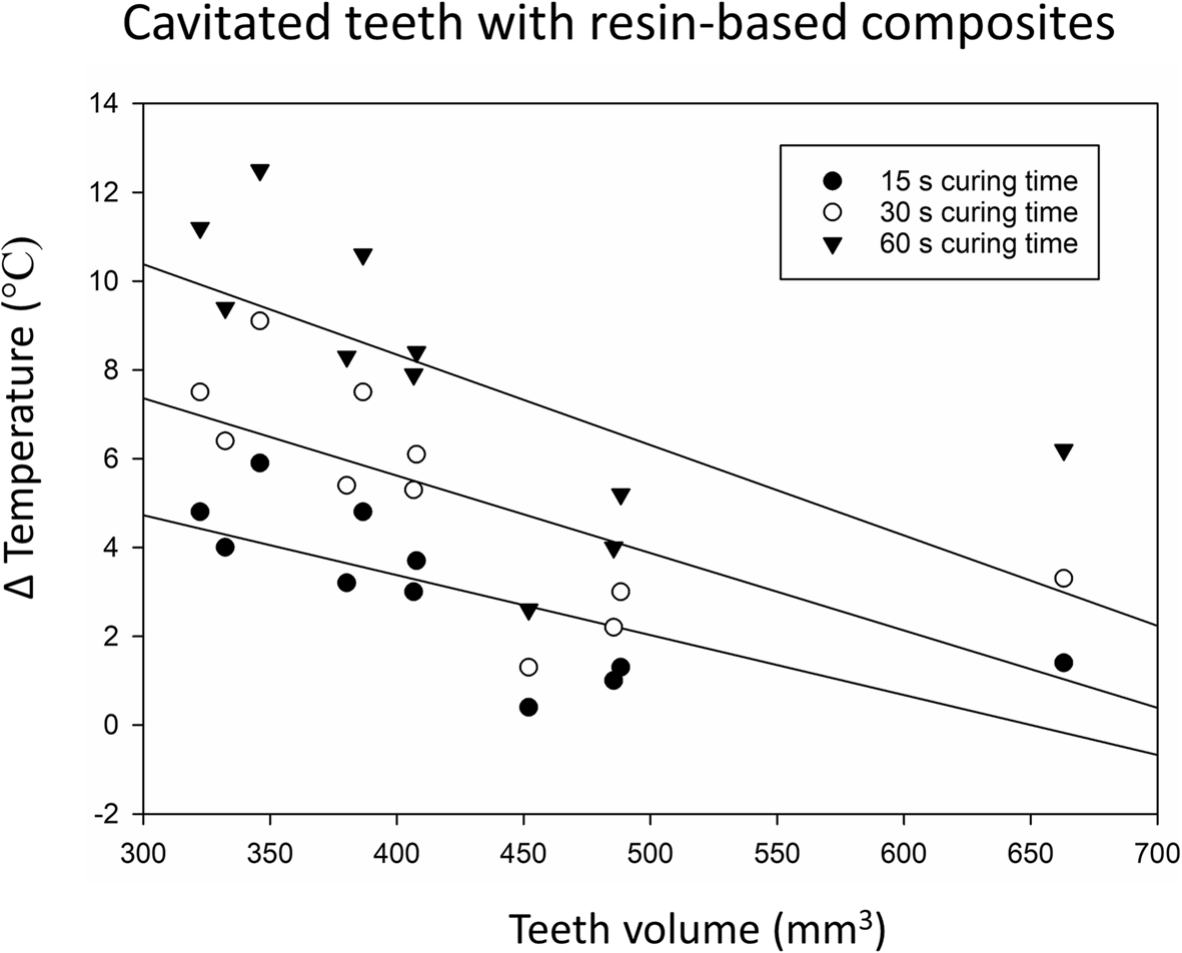
Relationship between pulp chamber temperature increase and the tooth volume for the teeth with resin-based composite (RBC)

Higher teeth volume corresponded to lower pulp chamber temperature increase for the time intervals of 15 s, 30 s and 60 s for the teeth with and without RBC (Table 2, Figs. 3 and 4).

According to generalized estimating equations, 10 mm^3^ increase in teeth volume resulted in 0.17 °C lower temperature increase in pulp chamber without air-cooling while it resulted in 0.04 lower temperature increase with air-cooling (β = − 0.17, 95%CI -0.30; − 0.05 and β = − 0.04, 95%CI -0.07; − 0.01, respectively) (Table 2).

## Discussion

### Temperature development in pulp chamber with and without air-cooling

This study showed that indirect air-cooling using three-way-syringe significantly decreased the temperature increase in the pulp chamber during light curing of teeth with and without RBC (confirming our first hypothesis). In relation to Zach and Cohens suggested temperature threshold for pulpal damage of 5.5 °C, in teeth with air-cooling the temperature increase did not exceed 4 °C (even after 60 s of LED-LCU exposure). In contrast, without air-cooling, in several teeth exposed for 30 s to LED-LCU and nearly in all teeth exposed for 60 s to LED-LCU, the pulp chamber temperature increase exceeded 5.5 °C.

The results of our study are in agreement with previous in vitro [24] and in vivo studies [25] demonstrating that air-cooling during LED-LCU exposure reduced the pulp chamber temperature increase. An in vitro study compared three cooling strategies (air-cooling, water and water spray) and concluded that air-cooling from the dental unit was the most efficient method to reduce temperature increase with LED-LCUs around an exposed tooth and within the pulp chamber during onlay luting [24]. Park and colleagues also reported that air-cooling is effective to reduce the maximum temperature reached within the pulp chamber. An embedded cooling system in a LED-LCU with a radiant emittance of 3000 mW/cm^2^ did not show greater maximum pulp chamber temperature reached after 60 s exposure time compared to a LED-LCU with a radiant emittance of 2000 mW/cm^2^. Moreover, since the cooling system remained activated after the LED-LCU was switched off, they showed that this increased the cooling speed [13]. An in vivo study from Zarpellon and co-workers reported that up to a 30 s LED-LCU exposure simultaneously with air-cooling did not increase the pulp chamber temperature [25]. This was not a case in our study, where median pulp chamber temperature increased by 1 °C in teeth without RBC and 0.4 °C in teeth with RBC with air-cooling after 30 s LED-LCU exposure. In the work from Zarpellon and others [25], air-cooling was started 3 s prior to the LED-LCU exposure while in our study air-cooling was started at the same instant as the LED-LCU. This might explain why in our case pulp chamber temperature increased already after 30 s of LED-LCU exposure. Therefore, a prior air-cooling of the tooth before LED-LCU exposure might be even more beneficial.

Of note, Park et al. [13] employed a “direct” air-cooling since it was installed on the LED-LCU unit while our study and other similar studies used an indirect air-cooling setup. The other methodological consideration that could influence pulp chamber temperature increase would be the location of the thermocouple inside the pulp chamber and the distance to the pulpal wall. In our study this parameter was carefully measured under high-resolution Micro-CT and the statistical analysis showed that the distance had no significant association with the pulp chamber temperature increase.

As part of the temperature regulation system for a tooth, the microcirculation is considered a contributing factor in the pulp chamber temperature [12]. In our study, the microcirculation system was not simulated. Thus, the chosen methodology is mimicking the clinical worst-case-scenario, e.g., where circulation is minimized using vasoconstrictor-containing local anesthesia.

### Pulp chamber temperature increase with air-cooling in teeth with RBC and without RBC

In our study, with air-cooling, the temperature increase in pulp chamber was statistically significantly lower in teeth with RBC than in teeth without RBC, confirming our second hypothesis. Pulp chamber temperature increase may be influenced by the translucency and thermal diffusivity of the substrate interposed between the heat source and the pulp [25]. Tetric EvoFlow® A3 shade (Ivoclar Vivadent, Schaan, Liechtenstein) was used in this work; it contains 57.5% in weight (30.7% in volume) barium glass, ytterbiumtrifluoride, mixed oxide and highly dispersed silica inorganic fillers [30]. Therefore, the findings may be different using other restorative materials. Moreover, it has been previously shown that polymerized RBC might have an insulating effect [15, 28]. In our study, temperature increase in pulp chamber was lower in teeth with RBC compared to teeth without RBC at 30 s and 60 s exposure time, suggesting that RBC may have an insulating effect. While at 15 s exposure time the temperature increase was higher in teeth with RBC, the exothermic reaction during the RBC polymerization might explain the higher temperature rise even though air-cooling is applied [5]. However, these differences were not statistically significant most likely because of a small sample size as only 11 teeth were included in our study. Studies with larger sample size are needed to investigate RBC and air-cooling interaction on pulp chamber temperature increase at different LED-LCU exposure times.

### Influence of the volume of teeth on pulp chamber temperature increase

Several in vitro studies observed temperature increase in pulp chamber ranging from 1.5 °C to 23.2 °C [1, 6, 9–14]. Such large range of temperature increase may be related to several factors including the differences in the volume of teeth investigated. In 2015, Runnacles and co-workers performed an in vivo study demonstrating that wide-spectrum LED-LCU increased temperature in the pulp chamber with some cases exceeding the threshold of 5.5 °C. This study was performed on young patients including only intact first upper premolars, which had similar volume [16]. In 1965, in an in vivo study performed in monkeys by applying a soldering iron on the tested teeth, Zach and Cohen found that a temperature increase of 5.5 °C in a healthy pulp resulted in necrosis in 15% of the teeth. It is worth to notice that the authors emphasized that pulps of “small teeth” were more likely to become necrotic [7]. To our knowledge, there are no studies that investigated the influence of the volume of the teeth on the pulp chamber temperature increase when exposed to LED-LCU. Our results showed that teeth with higher volume resulted in lower pulp chamber temperature increase. This finding confirms our third hypothesis demonstrating an association between tooth hard tissue volume and pulp chamber temperature increase during LED-LCU exposure.

Augmentation of 10 mm^3^ in teeth volume resulted in 0.17 °C lower temperature rise without air-cooling, while with air-cooling it resulted in 0.04 °C lower temperature rise. This finding suggests that lower volume teeth benefit more from the air-cooling than higher volume teeth. It might be of importance for incisors and young permanent teeth with large pulp chambers and thus lower volume of hard tooth tissue.

Teeth exposed to newly commercialized LED-LCU with ultra-high radiant emittance (up to 3000 mW/cm^2^) that have been shown to result in higher pulp chamber temperature increase might substantially benefit from indirect air-cooling. Moreover, many RBCs are placed in increments and are light cured in between each layer for up to 30 s. This might have an additive effect on pulp chamber temperature increase. Again, air-cooling might be an effective tool to mitigate the temperature increase in the pulp. There is sparse data available concerning this point and the subject is therefore potentially of interest for future studies.

In some clinical situations, where the amount of hard tissue or RBC between the LED-LCU and the pulp chamber is relatively thin, the pulp chamber temperature increase has been shown to be higher than in intact tooth [5]. Therefore, air-cooling used simultaneously with LED-LCU exposure might be a useful tool to mitigate pulp temperature increase in clinical scenarios, such as in class V preparation, bleaching that uses photo-activation or placement of composite resin veneers [25]. Several strategies have been proposed to reduce pulp chamber temperature development during RBC polymerization such as reduced irradiance power [21, 31] or modulated output mode [19, 32, 33]. However, these methods could lead to poorly cured RBC or increased time to achieve adequate polymerization. The use of air-cooling, which is easily available from the dental unit, might be a simple and efficient measure to mitigate temperature increase within the pulp chamber when using LED-LCU for RBC polymerization. There is a need for more studies investigating the effectiveness of air-cooling when using LED-LCUs, especially including lower volume teeth.

## Conclusion

Indirect air-cooling with the three-way-syringe resulted in lower pulp chamber temperature increase during light curing in teeth despite absence/presence of RBC restorative materials. Using air-cooling, the temperature increase was lower in teeth with RBC restorations compared to teeth without RBC. Lower coronal volume resulted in higher pulp chamber temperature increase, thus they seemed to benefit more from the air-cooling compared to higher volume teeth. AC could be an effective tool in controlling pulp temperature increase during LED-LCU exposure, especially when the tooth volume is small.

## Data Availability

The datasets analyzed during the current study are available from the corresponding author on reasonable request.

## Abbreviations

AC: Air-cooling
CI: Confidence intervals
IQR: Interquartile range
LCUs: Light-curing units
LED: Light-emitting diode
Micro-CT: Micro computed tomograph
RBC: Resin-based composite
REC: The Norwegian Regional committee for Medical and Health Research Ethics
s: Seconds

## References

[1] Leprince J, Devaux J, Mullier T, Vreven J, Leloup G (2010). Pulpal-temperature rise and polymerization efficiency of LED curing lights. Oper Dent.

[2] Price RB, Ferracane JL, Shortall AC (2015). Light-curing units: a review of what we need to know. J Dent Res.

[3] Todd J (2020). Scientific documentation 3s PowerCure.

[4] Ilie N, Watts DC (2020). Outcomes of ultra-fast (3 s) photo-cure in a RAFT-modified resin-composite. Dent Mater.

[5] Choi SH, Roulet JF, Heintze SD, Park SH (2014). Influence of cavity preparation, light-curing units, and composite filling on intrapulpal temperature increase in an in vitro tooth model. Oper Dent.

[6] Mouhat M, Mercer J, Stangvaltaite L, Ortengren U (2017). Light-curing units used in dentistry: factors associated with heat development-potential risk for patients. Clin Oral Investig.

[7] Zach L, Cohen G (1965). Pulp response to externally applied heat. Oral Surg Oral Med Oral Pathol.

[8] Baldissara P, Catapano S, Scotti R (1997). Clinical and histological evaluation of thermal injury thresholds in human teeth: a preliminary study. J Oral Rehabil.

[9] Eldeniz AU, Usumez A, Usumez S, Ozturk N (2005). Pulpal temperature rise during light-activated bleaching. J Biomed Mater Res B Appl Biomater.

[10] Yazici AR, Muftu A, Kugel G, Perry RD (2006). Comparison of temperature changes in the pulp chamber induced by various light curing units, in vitro. Oper Dent.

[11] Baroudi K, Silikas N, Watts DC (2009). In vitro pulp chamber temperature rise from irradiation and exotherm of flowable composites. Int J Paediatr Dent.

[12] Kodonas K, Gogos C, Tziafa C (2009). Effect of simulated pulpal microcirculation on intrachamber temperature changes following application of various curing units on tooth surface. J Dent.

[13] Park SH, Roulet JF, Heintze SD (2010). Parameters influencing increase in pulp chamber temperature with light-curing devices: curing lights and pulpal flow rates. Oper Dent.

[14] Oberholzer TG, Makofane ME, du Preez IC, George R (2012). Modern high powered led curing lights and their effect on pulp chamber temperature of bulk and incrementally cured composite resin. Eur J Prosthodont Restor Dent.

[15] Nilsen BW, Mouhat M, Haukland T, Örtengren UT, Mercer JB (2020). Heat development in the pulp chamber during curing process of resin-based composite using multi-wave LED light curing unit. Clin Cosmet Investig Dent.

[16] Runnacles P, Arrais CA, Pochapski MT, Dos Santos FA, Coelho U, Gomes JC (2015). In vivo temperature rise in anesthetized human pulp during exposure to a polywave LED light curing unit. Dent Mater.

[17] Rueggeberg FA, Giannini M, Arrais CAG, Price RBT (2017). Light curing in dentistry and clinical implications: a literature review. Braz Oral Res.

[18] Kwon SJ, Park YJ, Jun SH, Ahn JS, Lee IB, Cho BH (2013). Thermal irritation of teeth during dental treatment procedures. Restor Dent Endo.

[19] Huang TK, Hung CC, Tsai CC (2006). Reducing, by pulse width modulation, the curing temperature of a prototype high-power LED light curing unit. Dent Mater J.

[20] Hofmann N, Markert T, Hugo B, Klaiber B (2003). Effect of high intensity vs. soft-start halogen irradiation on light-cured resin-based composites. Part I. temperature rise and polymerization shrinkage. Am J Dent.

[21] Uhl A, Mills RW, Jandt KD (2003). Polymerization and light-induced heat of dental composites cured with LED and halogen technology. Biomaterials..

[22] Neo BJ, Soh MS, Teo JW, Yap AU (2005). Effectiveness of composite cure associated with different light-curing regimes. Oper Dent.

[23] Roulet JF, Price R (2014). Light curing - guidelines for practitioners - a consensus statement from the 2014 symposium on light curing in dentistry held at Dalhousie University, Halifax, Canada. J Adhes Dent.

[24] Onisor I, Asmussen E, Krejci I (2011). Temperature rise during photo-polymerization for onlay luting. Am J Dent.

[25] Zarpellon DC, Runnacles P, Maucoski C, Coelho U, Rueggeberg FA, Arrais C (2019). Controlling in vivo, human pulp temperature rise caused by LED curing light exposure. Oper Dent.

[26] Al-Qudah AA, Mitchell CA, Biagioni PA, Hussey DL (2005). Thermographic investigation of contemporary resin-containing dental materials. J Dent.

[27] Kim RJ, Lee IB, Yoo JY, Park SJ, Kim SY, Yi YA (2015). Real-time analysis of temperature changes in composite increments and pulp chamber during photopolymerization. Biomed Res Int.

[28] Yang J, Algamaiah H, Watts DC (2021). Spatio-temporal temperature fields generated coronally with bulk-fill resin composites: a thermography study. Dent Mater.

[29] Kopperud SE, Rukke HV, Kopperud HM, Bruzell EM (2017). Light curing procedures - performance, knowledge level and safety awareness among dentists. J Dent.

[30] Mirică IC, Furtos G, Bâldea B, Lucaciu O, Ilea A, Moldovan M (2020). Influence of filler loading on the mechanical properties of flowable resin composites. Materials..

[31] Ozturk B, Ozturk AN, Usumez A, Usumez S, Ozer F (2004). Temperature rise during adhesive and resin composite polymerization with various light curing sources. Oper Dent.

[32] Jo SA, Lee CH, Kim MJ, Ferracane J, Lee IB (2019). Effect of pulse-width-modulated LED light on the temperature change of composite in tooth cavities. Dent Mater.

[33] Hubbezoglu I, Dogan A, Dogan OM, Bolayir G, Bek B (2008). Effects of light curing modes and resin composites on temperature rise under human dentin: an in vitro study. Dent Mater J.

